# Network pharmacology exploration reveals a common mechanism in the treatment of cardio-cerebrovascular disease with *Salvia miltiorrhiza* Burge. and *Carthamus tinctorius* L

**DOI:** 10.1186/s12906-020-03026-y

**Published:** 2020-11-19

**Authors:** Yu Wang, Yajun Shi, Junbo Zou, Xiaofei Zhang, Yulin Liang, Jia Tai, Chunli Cui, Mei Wang, Dongyan Guo

**Affiliations:** 1 Department of Pharmaceutics, College of Pharmacy, Shaanxi University of Chinese Medicine, Xianyang, 712000 Shaanxi China; 2 Key Laboratory of basic and new drug research of traditional Chinese medicine, Shaanxi University of Chinese Medicine, Xianyang, 712000 Shaanxi China

**Keywords:** Cardiovascular agents, *Carthamus tinctorius*, Medicine, Traditional Chinese medicine, Myocardial infarction, Salvia miltiorrhiza

## Abstract

**Background:**

This study aimed to identify the key genes and KEGG pathways in *Carthamus tinctorius* L. (Safflower) and *Salvia miltiorrhiza* Burge. (Salvia) for the treatment of cardio-cerebrovascular disease, and to explore their potential molecular mechanisms.

**Methods:**

Compounds and targets in Safflower and Salvia were retrieved from Traditional Chinese Medicine Systems Pharmacology Database and Analysis Platform (TCMSP). We obtained targets of myocardial infarction (MI) and cerebral infarction (CI) data from Therapeutic Target Database (TTD), Drugbank and DisGeNET datasets. The network of Safflower, Salvia, CI and MI was established and then executing, and Kyoto Encyclopedia of Genes and Genomes (KEGG) and Gene Ontology (GO) analyses of the functional characteristics were performed. The Chinese herbal prescription and target for CI and MI were obtained by searching in the database. Finally, the main pathways of Salvia and Safflower in Chinese patent medicines were analyzed. The MCAO model was established in rats, and compatibility of salvia with safflower was experimentally verified.

**Results:**

We obtained a total of 247 genes targeted by 52 compounds from Safflower and 119 genes targeted by 48 compounds from Salvia. In total, we identified 299 known therapeutic targets for the treatment of CI and 960 targets for the treatment MI. There are 23 common targets for Salvia, Safflower, MI, and CI. A total of 85 KEGG pathways were also enriched and intersected with the pathway of proprietary Chinese medicine to yield 25 main pathways. Safflower and Salvia have the best therapeutic effect in MCAO.

**Conclusion:**

We identified gene lists for Safflower and Salvia in CI and MI. Bioinformatics and interaction analyses may provide new insight into the treatment of cardio-cerebrovascular diseases with Safflower and Salvia.

## Background

*Carthamus tinctorius* L. (Safflower) is a traditional Chinese medicine (TCM) used to promote blood circulation and remove blood stasis. There are many compounds in Safflower, including pigments, flavonoids, alkaloids, and organic acids, among others [1]. Among such compounds, Safflower yellow A and kaempferol are quality control indicators and also the main active compounds [2]. Safflower has multiple pharmacological activities that affect cardiovascular, cerebrovascular, circulatory, nervous, and immune systems, as well as anti-oxidant and anti-aging activities [3].

*Salvia miltiorrhiza* Burge. (Salvia) is a Chinese medicine that has a wide range of cardiovascular and cerebrovascular-protective activities [4]. The published literature indicates that the lipophilic components of Salvia include tanshinone I, tanshinone IIA, tanshinone IIB, cryptotanshinone, and dihydrotanshinone. The hydrophilic components include Danshensu, salvianolic acids A and B, and protocatechuic aldehyde [5, 6]. As a representative of Chinese medicine for cardio-cerebrovascular disease, Salvia has significant effects on myocardial protection, anti-atherosclerosis, anti-thrombosis, and microcirculation improvement. From in-depth studies of the pharmacological effects of Salvia, it has been found that it not only plays a protective role in the cardiovascular, but also plays a protective role in the nervous and digestive systems, which provide a theoretical basis for expanding its clinical use [7, 8].

Cardiovascular and cerebrovascular diseases are common in the clinic. Modern medicine holds that there is a close relationship between the heart and brain, which coordinate each other’s functions through nerve reflexes and humoral regulation. In clinical practice, atherosclerosis is often the common pathological basis of cardiovascular and cerebrovascular diseases. Hypertension, hyperlipidemia, smoking, and other risk factors of atherosclerosis and abnormal hemorheology also impact cardiovascular and cerebrovascular diseases. Therefore, lipid-lowering, blood pressure-controlling, blood viscosity-lowering drugs, and the rational use of drugs that not only treat cardiovascular and cerebrovascular, but also treat the two diseases simultaneously are needed [9]. Based on the holistic view of Chinese medicine, and guided by the theory of “treating the heart and brain together,” it is useful for Chinese medicine to leverage its advantages of imparting multiple approaches with multiple targets for the treatment and prevention of cardiovascular and cerebrovascular diseases [10, 11].

Safflower and Salvia are compatible and commonly paired in modern traditional Chinese medicine prescriptions. The two drugs function to activate blood circulation, remove blood stasis, and dredge collateral circulation in TCM theory. Both Safflower and Salvia have been used clinically for hundreds of years and remain popular. Therefore, it is necessary to identify the mechanisms by which Salvia and Safflower treat cerebral infarction (CI) and myocardial infarction (MI).

Studies have shown that the compatibility of Safflower with the main components of Salvia had therapeutic effects on MI [12]. The combination of water-soluble components of Safflower and Salvia was more effective in treating platelet aggregation and thrombosis induced by ischemia-reperfusion injury in the myocardium and cerebral ischemia-reperfusion injury in rats than either Salvia or Safflower alone [13]. The effective fractions of Safflower and Salvia extracts (salvianolic acid and Safflower yellow pigment) were made compatible by optimizing the components of traditional Chinese medicine and effectively alleviated myocardial injury, inhibited thrombosis, and protected and improve myocardial ischemia in rats with myocardial ischemia and reperfusion [14].

Currently, there are many Chinese patent medicines compatible with Safflower and Salvia [15]. The compatibility application of Safflower and Salvia being used mostly in modern prescriptions and rare in ancient prescriptions. Due to those problems, a proprietary Chinese medicine approach containing Safflower and Salvia was assessed using bibliometrics [15]. Multiple Chinese patent medicines exist, including Xin Naokang capsules, Le Mai granules, Li Nao Xin capsules, Huoxue Tongmai tablets, Danhong dripping solutions, and Guan Xin Jing capsules, among others [16].

**Fig. 1:**
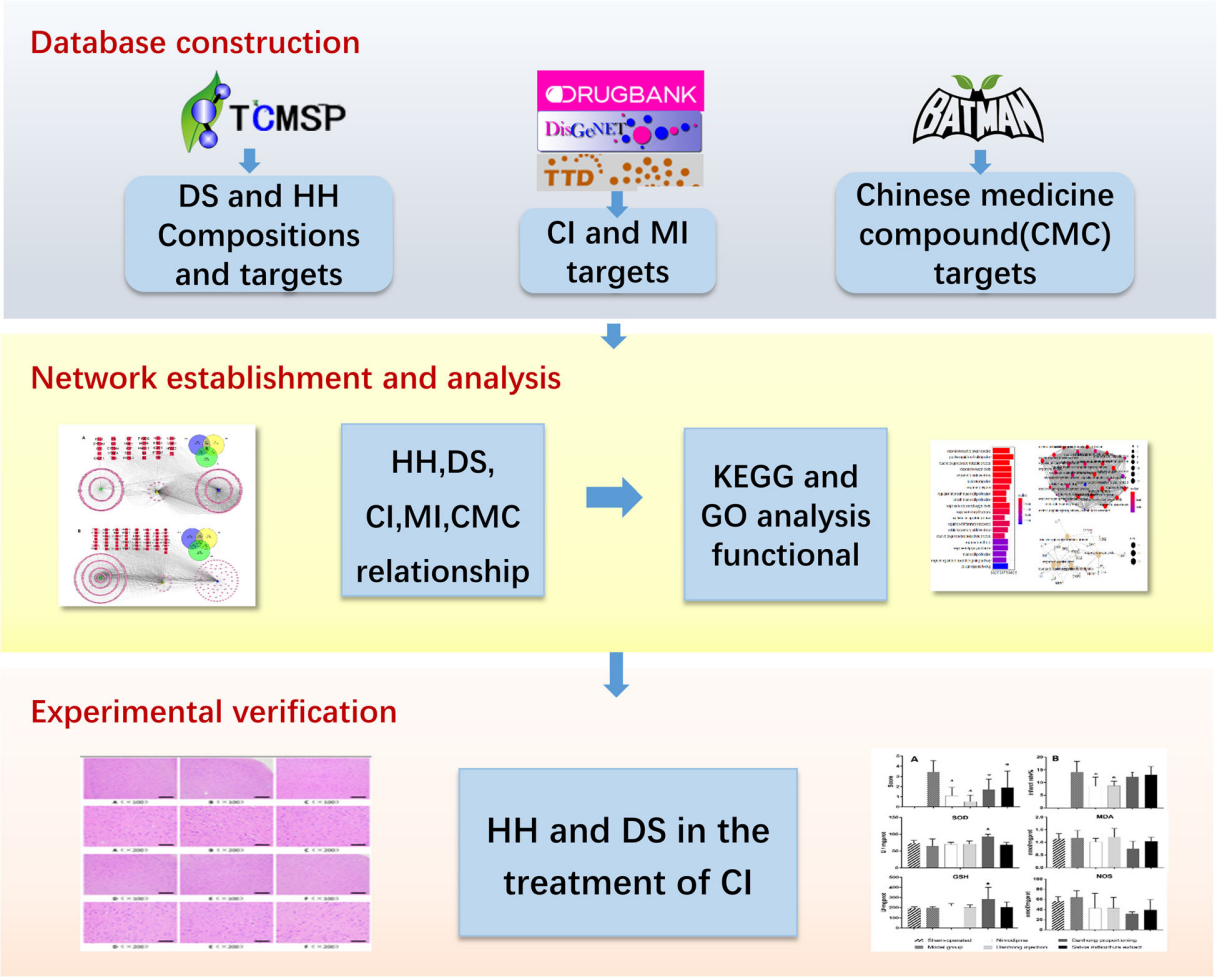
Workflow for Salvia and Safflower co-treatment of CI and MI. HH, *Carthamus tinctorius* L. DS, *Salvia miltiorrhiza* Burge. MI, myocardial infarction. CI,cerebral infarction

Recently, the development of network pharmacology has provided a new approach to investigate the compatibility of TCM compounds and TCM. This study aimed to explore the mechanisms of Safflower and Salvia compounds for the treatment of MI and CI using network pharmacology (Fig. 1).

## Methods

### Data preparation

#### Construction of the chemical information database for salvia and safflower

The molecular information of the main components of Salvia and Safflower was obtained by searching the TCMSP database (http://lsp.nwu.edu.cn/tcmsp.php). TCMSP is a unique pharmacology platform for Chinese herbal medicines that capture the relationships between drugs, targets, and diseases. The database includes chemicals, targets, and drug-target networks, as well as associated with drug-target-disease networks and the pharmacokinetics properties for natural compounds with oral bioavailability, drug-likeness, intestinal epithelial permeability, blood-brain-barrier, and aqueous solubility. This breakthrough has sparked new interest in the search for candidate drugs from various types of traditional Chinese herbs.

#### Known therapeutic drug targets for the treatment of MI and CI

The known therapeutic targets of drugs used to treat CI and MI were acquired from two sources: the DrugBank (http://www.drugbank.ca/, version 4.3) and the TTD database (https://db.idrblab.org/ttd/, updated on Sep 15, 2017). Those databases provide information about known and explored therapeutic proteins and nucleic acid targets, target diseases, pathway information, and the drugs directed at each of those targets. DisGeNET (https://www.disgenet.org/) is a versatile platform that can be used for different research purposes, including investigations of the molecular underpinnings of human diseases and their comorbidities, analyses of the properties of disease genes, the generation of hypotheses on the therapeutic actions and adverse effects of drugs, the validation of computationally predicted disease genes, and the evaluation of text-mining methods.

#### Prediction of putative targets of chemical composition

The chemical components of each prescription were collected from BATMAN-TCM (http://bionet.ncpsb.org/batman-tcm/). BATMAN-TCM (a Bioinformatics Analysis Tool for Molecular mechANism of Traditional Chinese Medicine) is the first online bioinformatics analysis tool specifically designed for research into the molecular mechanisms of TCM.

A drug similarity search tool, ChemMapper (http://lilab.ecust.edu.cn/chemmapper/), was effective at rapidly identifying all molecules with structural similarities, and was used to identify known drugs that are structurally similar to chemical components. The therapeutic targets of the drugs obtained from the DrugBank database were considered as the putative drug targets.

### Network construction and analysis

With the popularization of system biology, network pharmacology based on big data has become an important method to analyze the action mechanism of complex traditional Chinese medicine prescriptions. The disease-target-drug network was constructed to understand the associations between herbs and chemical compounds, the putative targets of Safflower and Salvia, and the known therapeutic targets for MI and CI. Network visualization was performed using Cytoscape software (version 3.2.1, USA). All node degrees of the network were calculated at the same time.

### Functional enrichment analysis

To make a better understanding of the underlying biological processes and pathways of the network, GO and KEGG pathway analyses were performed using the clusterProfiler package [17] in R platform. Following this, the R topGO package [18] was employed to reconstruct the GO interaction network.

### Preparation of extracts of salvia and safflower

Salvia was extracted with water at 80 °C for 120 min. After extracting twice, the liquid is combined and filtered. The filtrate was vacuum concentration at 60 °C to a relative density of 1.18 to 1.22 (refer to the concentration and drying process in Chinese Pharmacopoeia). Then add ethanol to make it contain 70% alcohol, and let it stand for 12 h. After taking the supernatant, the ethanol was recovered and concentrated into a thick paste. The extract of Salvia was obtained by drying in a vacuum drying box (the yield was 10%).

Safflower was extracted with water at 90 °C for 60 min. After extracting twice, the liquid is combined and filtered. The filtrate was concentrated to thick paste under decompression at 60 °C (refer to the concentration and drying process in Pharmacopoeia). The extract of Safflower was obtained by drying in a vacuum drying box (the yield was 35%).

The extract of Salvia and Safflower of the same quality was mixed to form the Danhong compatibility group.

### Animal experiment to verify the efficacy

Male Sprague-Dawley rats (250 ± 25 g) were used and supplied by the Xi’an Jiaotong University Medical Laboratory Animal Center (Xi’an, China). The study was approved by the Ethical Committee of the Shaanxi University of Chinese Medicine. After 1 week of feeding, 96 rats were randomly divided into 6 groups: Sham operation group, Model group, Nimodipine group, Danhong injection group, Danhong compatibility group, Salvia extract group. In the Middle cerebral artery occlusion (MCAO) model, the mortality rate was high, so each group of experimental animals was set at 16 animals. The dose of intranasal administration was 0.1 ml per rat.

On the 8th day, the MCAO model was used to establish the rat model of focal cerebral ischemia. The rats were anesthetized with 10% chloral hydrate (3 mL/kg) by intraperitoneal injection before Operation. After 2 h of cerebral ischemia and 3 h of reperfusion, the neurobehavioral Longa score was used. The rats were anesthetized with 1% Pentobarbital Sodium for euthanasia. Blood was taken from the abdominal aorta of rats for 5 mL, and then decapitate the brain and weigh it. Tetrazole red (TTC) staining was used to determine the cerebral infarction rate. The whole brain of the rat was placed in a small bottle containing neutral formalin. The brain tissue was sliced and the morphology of the cells was observed by hematoxylin-eosin staining.

T-test was used to evaluate the data of various indicators and the statistical differences between the treatment groups and the model group.

## Results

### Putative targets of safflower and salvia

We obtained a total of 189 constituent compounds from Safflower and 202 constituent compounds from Salvia. After screening using a TCMSP DL-score of more than 0.18, a total of 247 genes targeted with 52 compounds such as Safflow-yellow-A, Carthamone, Kaempferol, Rutin, Nicotiflorin, Luteolin, 6-hydroxykaempferol-3-O-glucoside, Precarthamin, Safflomin-C, Hydroxysafflor-yellow-A, Octacosane in Safflower were obtained. After screening using a TCMSP drug-likeness (DL) score of more than 0.18, an oral bioavailability (OB) score of more than 30%, and a half-life (HL) of more than 4 h, a total of 119 genes targeted with 48 compounds such as Tanshinone II A, Tanshinone VI, Tanshindiol B, Dehydrotanshinone II A, 4-methylenemiltirone, Formyltanshinone, Danshenol A, Isotanshinone II, Miltionone I, Prolithospermic acid in Salvia were obtained [19]. Detailed information on the constituent compounds for each herb is provided in Table 1.

**Table 1:** Characterization and details information in 52 safflower compounds and 48 salvia compounds. OB: oral bioavailability. DL: drug-like

ID	Compound	OB/%	DL	ID	Compound	OB/%	DL
HH-01	Syrigin	14.64	0.32	DS-01	1,2,5,6-tetrahydrotanshinone	38.75	0.36
HH-02	Pyrethrin II	48.36	0.35	DS-02	Poriferasterol	43.83	0.76
HH-03	VIV	14.26	0.55	DS-03	Poriferast-5-en-3beta-ol	36.91	0.75
HH-04	Gamma-Tocotrienol	20.3	0.53	DS-04	Sugiol	36.11	0.28
HH-05	Rutin	3.2	0.68	DS-05	Dehydrotanshinone II A	43.76	0.4
HH-06	6-hydroxykaempferol-3-O-beta-D-glucoside	1.85	0.76	DS-06	Baicalin	40.12	0.75
HH-07	β-amyrin acetate	9.11	0.74	DS-07	Digallate	61.85	0.26
HH-08	Nicotiflorin	3.64	0.73	DS-08	Luteolin	36.16	0.25
HH-09	(+)-Syringaresinol	3.29	0.72	DS-09	5,6-dihydroxy-7-isopropyl-1,1-dimethyl-2,3-dihydrophenanthren-4-one	33.77	0.29
HH-10	Baicalin	40.12	0.75	DS-10	2-isopropyl-8-methylphenanthrene-3,4-dione	40.86	0.23
HH-11	6-Hydroxykaempferol	62.13	0.27	DS-11	3α-hydroxytanshinoneIIa	44.93	0.44
HH-12	Sitogluside	20.63	0.62	DS-12	(E)-3-[2-(3,4-dihydroxyphenyl)-7-hydroxy-benzofuran-4-yl]acrylic acid	48.24	0.31
HH-13	Astragalin	14.03	0.74	DS-13	4-methylenemiltirone	34.35	0.23
HH-14	Baicalein	33.52	0.21	DS-14	2-(4-hydroxy-3-methoxyphenyl)-5-(3-hydroxypropyl)-7-methoxy-3-benzofurancarboxaldehyde	62.78	0.4
HH-15	Luteolin	36.16	0.25	DS-15	Formyltanshinone	73.44	0.42
HH-16	Arachic acid	16.66	0.19	DS-16	3-beta-Hydroxymethyllenetanshiquinone	32.16	0.41
HH-17	Kaempferol	41.88	0.24	DS-17	Methylenetanshinquinone	37.07	0.36
HH-18	Apigenin	23.06	0.21	DS-18	Przewaquinone B	62.24	0.41
HH-19	Myricetin	13.75	0.21	DS-19	Przewaquinone c	55.74	0.4
HH-20	Scutellarein	18.97	0.24	DS-20	(6S,7R)-6,7-dihydroxy-1,6-dimethyl-8,9-dihydro-7H-naphtho [8,7-g] benzofuran-10,11-dione	41.31	0.45
HH-21	Lupeol	12.12	0.78	DS-21	Przewaquinone f	40.31	0.46
HH-22	Lignan	43.32	0.65	DS-22	Sclareol	43.67	0.21
HH-23	Nonacosanol	10.57	0.43	DS-23	Tanshinaldehyde	52.47	0.45
HH-24	Thymopentin	1.24	0.46	DS-24	Danshenol B	57.95	0.56
HH-25	Beta-carotene	37.18	0.58	DS-25	Danshenol A	56.97	0.52
HH-26	Quercetin	46.43	0.28	DS-26	Salvilenone	30.38	0.38
HH-27	ADO	15.98	0.18	DS-27	Cryptotanshinone	52.34	0.4
HH-28	Fluoranthene	24.7	0.18	DS-28	Dan-shexinkum d	38.88	0.55
HH-29	CLR	37.87	0.68	DS-29	Danshenspiroketallactone	50.43	0.31
HH-30	Poriferast-5-en-3beta-ol	36.91	0.75	DS-30	Deoxyneocryptotanshinone	49.4	0.29
HH-31	Beta-sitosterol	36.91	0.75	DS-31	Dihydrotanshinlactone	38.68	0.32
HH-32	Stigmasterol	43.83	0.76	DS-32	DihydrotanshinoneI	45.04	0.36
HH-33	Vitamin-G	6.79	0.50	DS-33	Isocryptotanshi-none	54.98	0.39
HH-34	Carthamone	5.93	0.63	DS-34	Isotanshinone II	49.92	0.4
HH-35	Quercetagetin	45.01	0.31	DS-35	Manool	45.04	0.2
HH-36	Hydroxysafflor-yellow-A	4.77	0.68	DS-36	Miltionone I	49.68	0.32
HH-37	Amoenin A3	3.32	0.74	DS-37	Miltirone	38.76	0.25
HH-38	Sesquiterpene	5.86	0.67	DS-38	Neocryptotanshinone II	39.46	0.23
HH-39	6-hydroxykaempferol-3-O-glucoside	1.97	0.76	DS-39	Neocryptotanshinone	52.49	0.32
HH-40	7,8-dimethyl-1H-pyrimido[5,6-g]quinoxaline-2,4-dione	45.75	0.19	DS-40	1-methyl-8,9-dihydro-7H-naphtho [5,6-g] benzofuran-6,10,11-trione	34.72	0.37
HH-41	5,8-dimethyltocol	15.62	0.52	DS-41	Prolithospermic acid	64.37	0.31
HH-42	6-hydroxykaempferol-3,6-di-O-beta-D-glucoside	4.21	0.68	DS-42	Salvianolic acid j	43.38	0.72
HH-43	Tagetiin	28.34	0.78	DS-43	(6S)-6-hydroxy-1-methyl-6-methylol-8,9-dihydro-7H-naphtho[8,7-g]benzofuran-10,11-quinone	75.39	0.46
HH-44	Sophoraflavonoloside	5.3	0.71	DS-44	Tanshindiol B	42.67	0.45
HH-45	Quercetin-3,7-di-O-beta-d-glucoside	5.86	0.67	DS-45	Przewaquinone E	42.85	0.45
HH-46	Lirioresinol-A	1.76	0.67	DS-46	Tanshinone IIA	49.89	0.4
HH-47	Precarthamin	22	0.67	DS-47	(6S)-6-(hydroxymethyl)-1,6-dimethyl-8,9-dihydro-7H-naphtho[8,7-g]benzofuran-10,11-dione	65.26	0.45
HH-48	Carthamone	5.93	0.63	DS-48	Tanshinone VI	45.64	0.3
HH-49	Octacosane	8.15	0.37	HH-51	Safflomin-C	5.57	0.66
HH-50	Safflomin-A	3.53	0.68	HH-52	Safflow-yellow-A	27.16	0.70

### Known therapeutic drug targets for the treatment of MI and CI

The known therapeutic targets for drugs used to treat MI and CI were acquired from two sources. Human disease-related genes were searched using the keywords “myocardial infarction,” “cerebral infarction,” and “*Homo sapiens*.” In total, we identified 316 known therapeutic targets for the treatment of CI and 999 targets for the treatment of MI. Detailed information about those targets is provided in Table S1. After removing redundant entries, 299 therapeutic targets for the treatment of CI and 960 targets for the treatment of MI were used for data analysis.

### Network construction and analysis

The network approach was applied to analyze Safflower and Salvia compounds and their corresponding targets, and to dissect the molecular mechanism of action of such compounds, from a network modulation point of view. Putative target networks were constructed to understand the associations between herbs and compounds in Safflower and Salvia, the putative targets of Safflower and Salvia, and the known therapeutic targets for MI and CI.

### Compound-target network for CI and MI

**Fig. 2:**
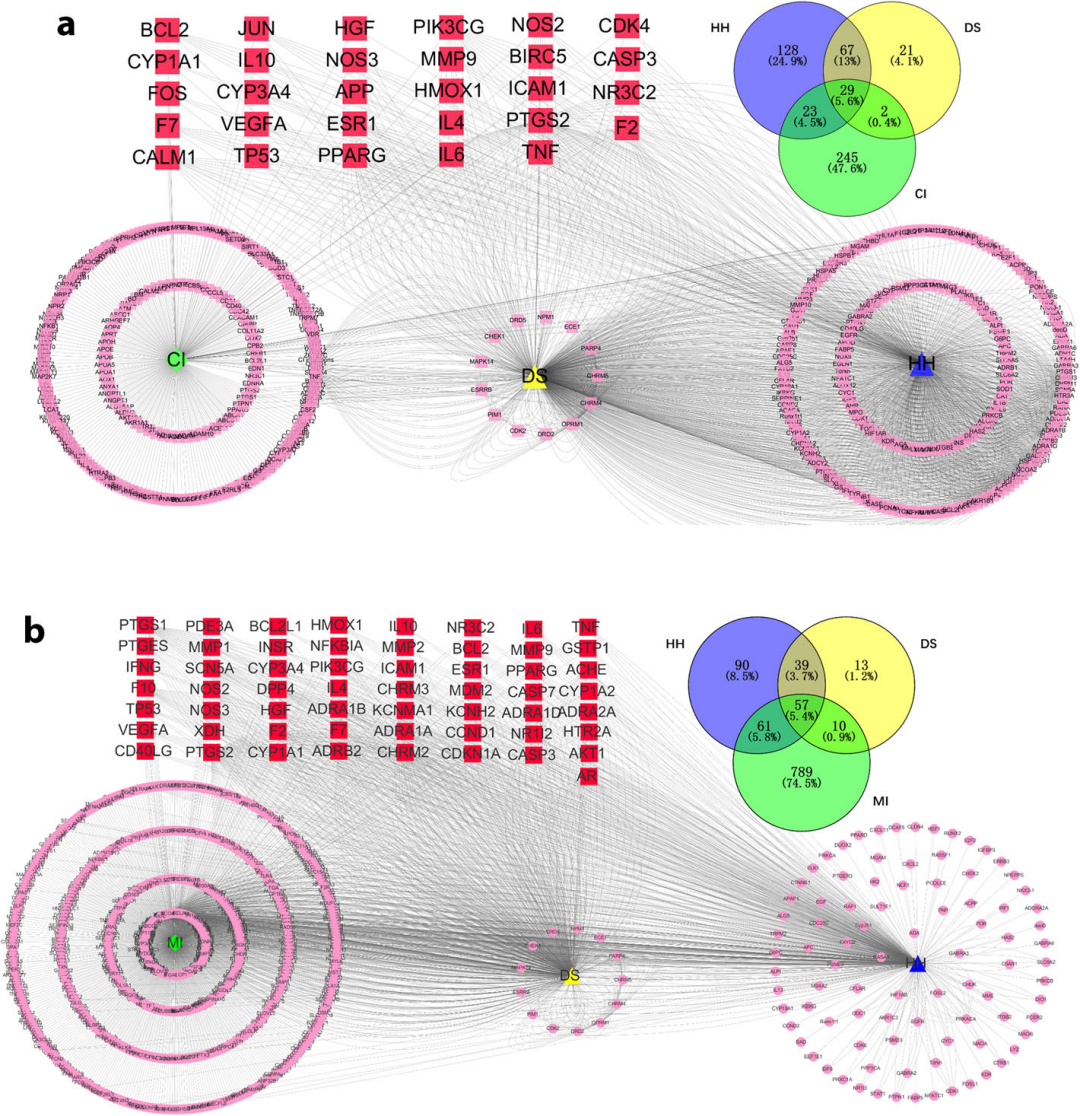
**a** Common targets of Safflower (blue) and Salvia (yellow) compounds for the treatment of CI (green). The network predicted 29 unique targets (red) related to CI. **b** Common targets of Safflower (blue) and Salvia (yellow) compounds for the treatment of MI (green). The network predicted 57 unique targets (red) related to MI. The pink circles represent target proteins (Venn diagrams showing the number of shared and unique targets by CI and MI)

The network diagram is shown in Fig. 2.A total of 29 targets were identified in Cytoscape from the 299 CI-related targets, 247 Safflower-related targets, and 119 Salvia-related targets (Fig. 2a). A total of 57 targets were also identified from 960 MI-related targets, 247 Safflower-related targets, and 119 Salvia-related targets (Fig. 2b).

**Fig. 3:**
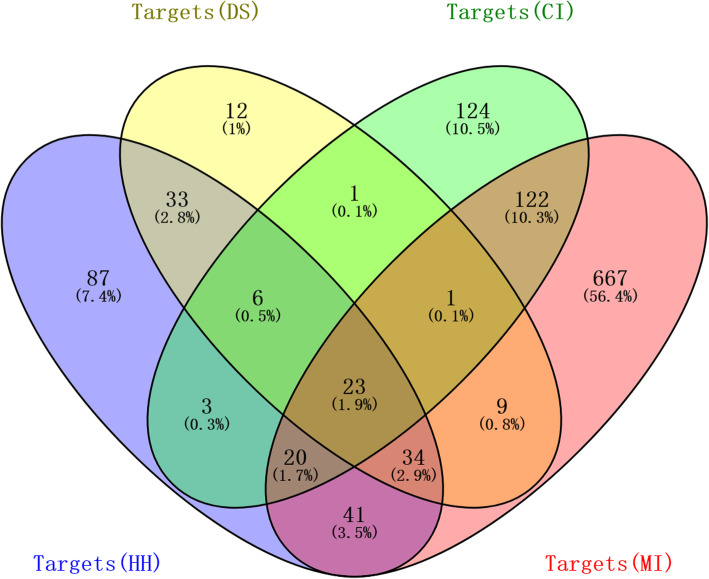
Venn diagram of common targets for Salvia and Safflower compounds in the treatment of MI and CI

The Venn graph can represent not only an independent set, but also the relationship between the set and the set. First of all, we use the Venn diagram to intersect the collected targets of diseases and medicinal materials. The central area of the picture is the intersection of the medicinal material and the target. Twenty-three such targets were common for MI and CI (Fig. 3), and were the focus of the following analyses, including PTGS2, NR3C2, F2, ESR1, PPARG, PIK3CG, VEGFA, MMP9, IL10, TNF, IL6, CASP3, TP53, HMOX1, ICAM1, IL4, HGF, NOS3, NOS2, F7, BCL2, CYP3A4, CYP1A1.

### Functional enrichment analysis of safflower and salvia in MI and CI

To understand the mechanisms involved in the development of MI and CI, the ClusterProfiler package in R was used to execute KEGG and GO analyses of the functional characteristics of Safflower and Salvia compounds [14]. Eighty-five KEGG pathways were enriched. The top 20 most enriched pathways are listed in Table 2.Fluid shear stress and atherosclerosis, the AGE-RAGE signaling pathway in diabetic complications, the IL-17 signaling pathway, malaria, the HIF-1 signaling pathway, the TNF signaling pathway, toxoplasmosis, Kaposi sarcoma-associated herpesvirus infection, proteoglycans in cancer, and leishmaniasis were involved in the pathological development of MI and CI.

**Table 2:** Top 20 enriched KEGG pathways of Safflower and Salvia compounds in MI and CI samples

Pathway names	***P*** value	p.adjust	qvalue	count
Fluid shear stress and atherosclerosis	4.55E-09	7.19E-07	3.11E-07	8
AGE-RAGE signaling pathway in diabetic complications	1.19E-08	9.44E-07	4.09E-07	7
IL-17 signaling pathway	2.69E-07	1.20E-05	5.19E-06	6
Malaria	3.03E-07	1.20E-05	5.19E-06	5
HIF-1 signaling pathway	4.16E-07	1.31E-05	5.69E-06	6
TNF signaling pathway	7.32E-07	1.83E-05	7.93E-06	6
Toxoplasmosis	8.58E-07	1.83E-05	7.93E-06	6
Kaposi sarcoma-associated herpesvirus infection	9.27E-07	1.83E-05	7.93E-06	7
Proteoglycans in cancer	1.56E-06	2.75E-05	1.19E-05	7
Leishmaniasis	2.44E-06	3.85E-05	1.67E-05	5
Pertussis	2.78E-06	4.00E-05	1.73E-05	5
African trypanosomiasis	3.36E-06	4.33E-05	1.87E-05	4
Hepatitis B	3.56E-06	4.33E-05	1.87E-05	6
PI3K-Akt signaling pathway	6.16E-06	6.95E-05	3.01E-05	8
Small cell lung cancer	7.57E-06	7.97E-05	3.45E-05	5
Amoebiasis	8.84E-06	8.73E-05	3.78E-05	5
Tuberculosis	1.25E-05	0.000116364	5.04E-05	6
Amyotrophic lateral sclerosis (ALS)	1.55E-05	0.000136143	5.90E-05	4
MicroRNAs in cancer	2.16E-05	0.000179924	7.79E-05	7
Inflammatory bowel disease (IBD)	4.08E-05	0.000322609	0.000139704	4

**Fig. 4:**
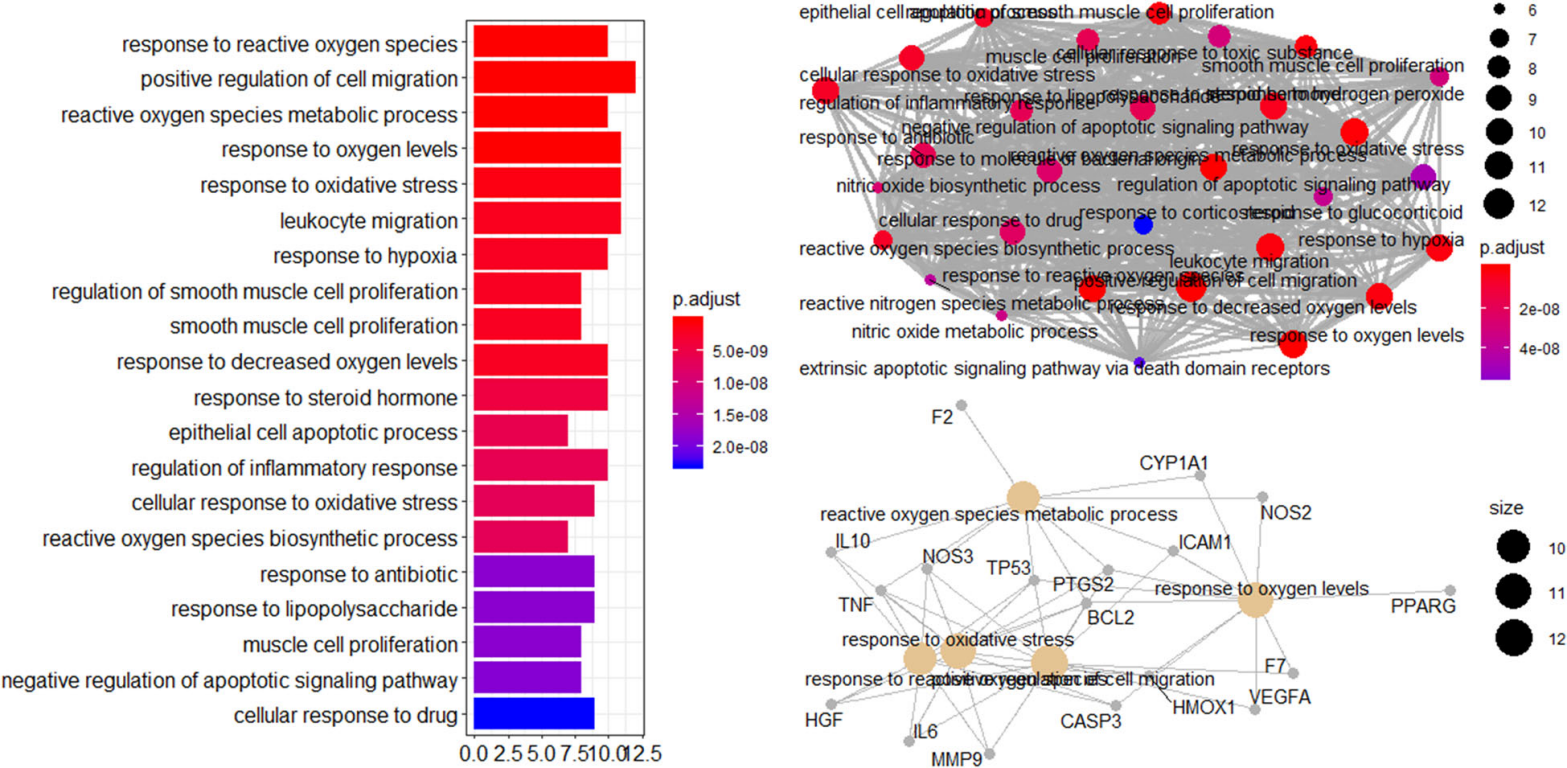
The 20 enriched GO terms (Biological Process) and GO interaction network with the most significant p-values for Safflower and Salvia in MI and CI

GO analyses revealed 2193 enriched GO terms in the “Biological Process (BP),” including responses to reactive oxygen species, positive regulation of cell migration, reactive oxygen species metabolic process, response to oxygen levels, response to oxidative stress, leukocyte migration, response to hypoxia, regulation of smooth muscle cell proliferation, smooth muscle cell proliferation, response to decreased oxygen levels, etc. (Table 3). By analyzing Fig. 4, we can clearly see that PTGS2, TNF, NOS3, IL6, BCL2, IL10, TP53, CASP3, HGF, and MMP9 are important hubs in the pathway.

**Table 3:** Enriched GO (BP) pathways of Safflower and Salvia compounds in MI and CI samples

Description	***P*** value	p.adjust	qvalue	count
Response to reactive oxygen species	1.00E-13	1.87E-10	6.09E-11	10
Positive regulation of cell migration	1.70E-13	1.87E-10	6.09E-11	12
Reactive oxygen species metabolic process	3.35E-13	2.21E-10	7.22E-11	10
Response to oxygen levels	4.03E-13	2.21E-10	7.22E-11	11
Response to oxidative stress	1.91E-12	8.36E-10	2.73E-10	11
Leukocyte migration	4.30E-12	1.57E-09	5.13E-10	11
Response to hypoxia	6.71E-12	1.84E-09	6.02E-10	10
Regulation of smooth muscle cell proliferation	6.73E-12	1.84E-09	6.02E-10	8
Smooth muscle cell proliferation	7.97E-12	1.84E-09	6.02E-10	8
Response to decreased oxygen levels	8.41E-12	1.84E-09	6.02E-10	10
Response to steroid hormone	2.24E-11	4.47E-09	1.46E-09	10
Epithelial cell apoptotic process	3.20E-11	5.86E-09	1.91E-09	7
Regulation of inflammatory response	3.67E-11	6.18E-09	2.02E-09	10
Cellular response to oxidative stress	4.54E-11	6.66E-09	2.17E-09	9
Reactive oxygen species biosynthetic process	4.55E-11	6.66E-09	2.17E-09	7
Response to antibiotic	1.46E-10	1.87E-08	6.10E-09	9
Response to lipopolysaccharide	1.50E-10	1.87E-08	6.10E-09	9
Muscle cell proliferation	1.53E-10	1.87E-08	6.10E-09	8
Negative regulation of apoptotic signaling pathway	1.65E-10	1.91E-08	6.23E-09	8
Cellular response to drug	2.12E-10	2.32E-08	7.57E-09	9

**Fig. 5:**
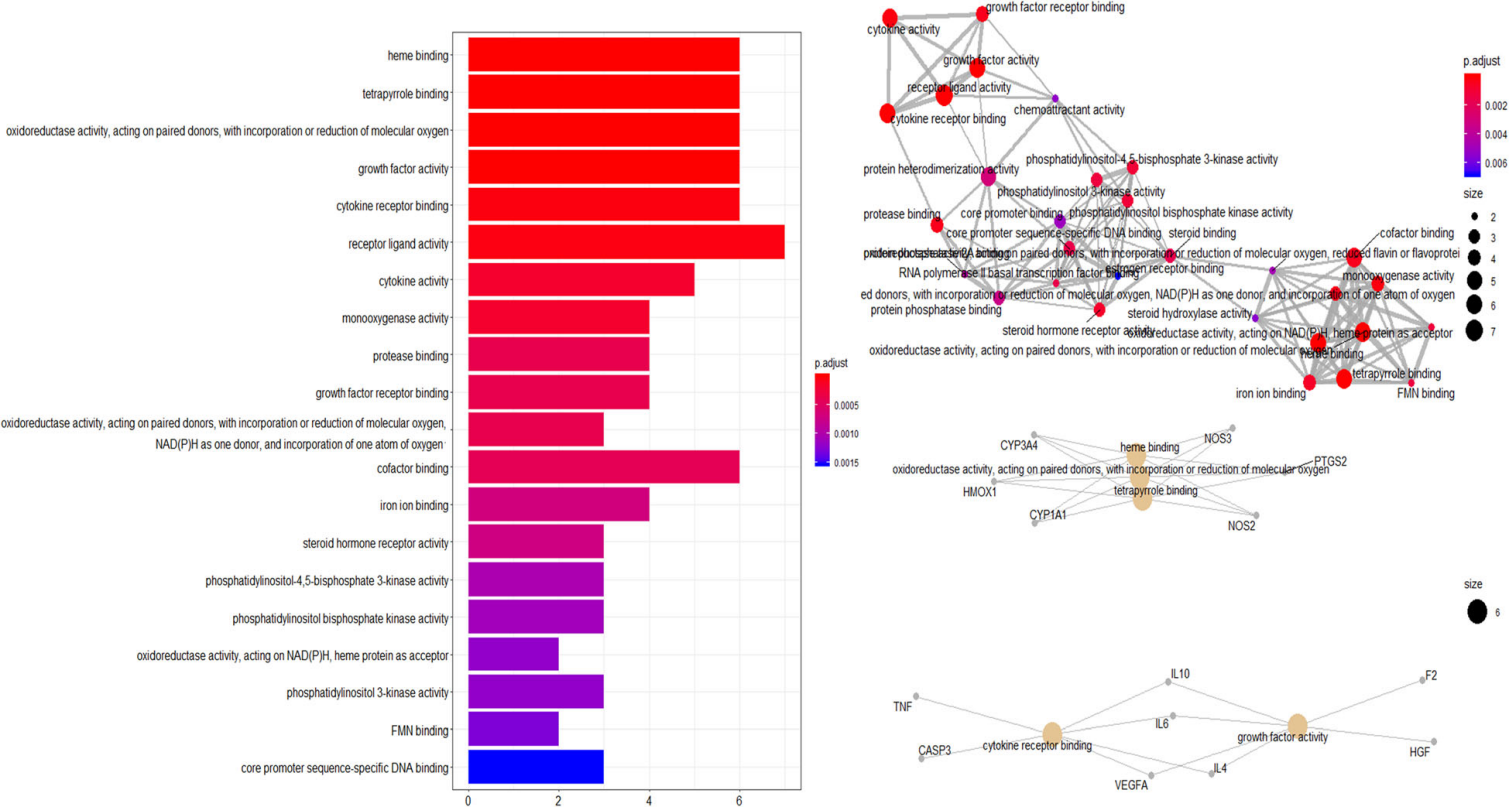
The 20 enriched GO terms (Molecular Function) and GO interaction network with the most significant *p*-values for Safflower and Salvia in MI and CI

GO analyses revealed 66 enriched GO terms in the “Molecular Function (MF),” including heme binding, tetrapyrrole binding, oxidoreductase activity, growth factor activity, cytokine receptor binding, receptor ligand activity, cytokine activity, monooxygenase activity, protease binding, growth factor receptor binding, etc. (Table 4). As shown in Fig. 5, combined with two network diagrams, we can see that PTGS2, NOS3, NOS2, CYP3A4, HMOX1, CYP1A1, IL10, IL6, IL4, and VEGFA play important roles in the pathway.

**Table 4:** Enriched GO (MF) pathways of Safflower and Salvia compounds in MI and CI samples

Description	***P*** value	p.adjust	qvalue	count
Heme binding	1.34E-08	1.74E-06	6.82E-07	6
Tetrapyrrole binding	2.09E-08	1.74E-06	6.82E-07	6
Oxidoreductase activity, acting on paired donors, with incorporation or reduction of molecular oxygen	4.64E-08	2.15E-06	8.46E-07	6
Growth factor activity	5.19E-08	2.15E-06	8.46E-07	6
Cytokine receptor binding	1.21E-06	4.00E-05	1.57E-05	6
Receptor ligand activity	1.64E-06	4.55E-05	1.79E-05	7
Cytokine activity	8.28E-06	0.000175	6.87E-05	5
Monooxygenase activity	8.42E-06	0.000175	6.87E-05	4
Protease binding	2.50E-05	0.00038	0.000149	4
Growth factor receptor binding	2.50E-05	0.00038	0.000149	4
Oxidoreductase activity, acting on paired donors, with incorporation or reduction of molecular oxygen, NAD(P)H as one donor, and incorporation of one atom of oxygen	2.52E-05	0.00038	0.000149	3
Cofactor binding	3.10E-05	0.000428	0.000168	6
Iron ion binding	5.40E-05	0.00069	0.000271	4
Steroid hormone receptor activity	6.09E-05	0.000723	0.000284	3
Phosphatidylinositol-4,5-bisphosphate 3-kinase activity	9.32E-05	0.001032	0.000406	3
Phosphatidylinositol bisphosphate kinase activity	0.000106	0.0011	0.000433	3
Oxidoreductase activity, acting on NAD(P)H, heme protein as acceptor	0.000127	0.001198	0.000471	2
Phosphatidylinositol 3-kinase activity	0.00013	0.001198	0.000471	3
FMN binding	0.000148	0.001294	0.000509	2
Heme binding	1.34E-08	1.74E-06	6.82E-07	6

**Fig. 6:**
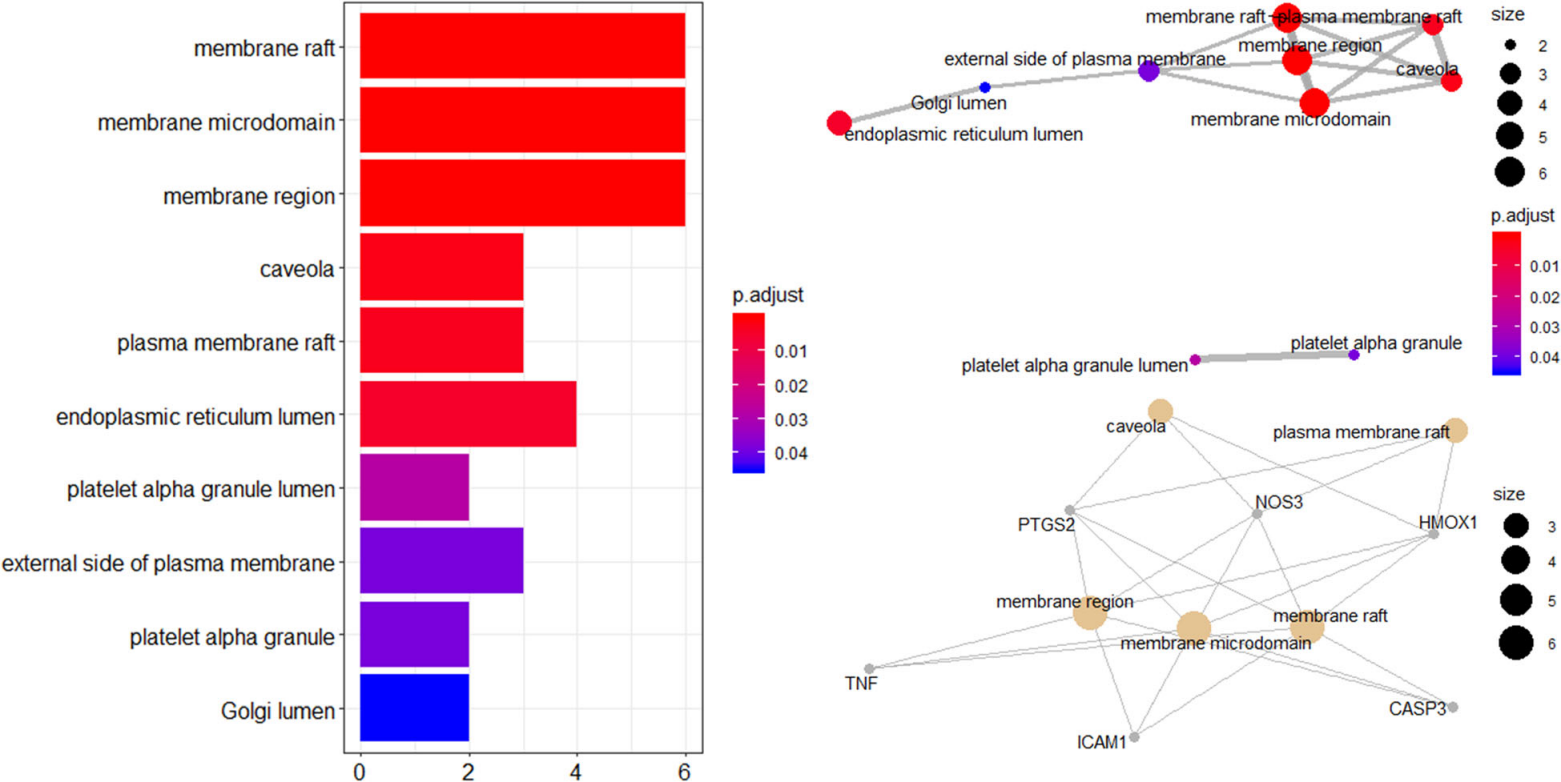
The 10 enriched GO terms (Cellular Component) and GO interaction network with the most significant p-values for Safflower and Salvia in MI and CI

GO analyses revealed 10 enriched GO terms in the “Cellular Component (CC),” including membrane raft, membrane microdomain, membrane region, caveola, plasma membrane raft, endoplasmic reticulum lumen, platelet alpha granule lumen, external side of plasma membrane, platelet alpha granule, Golgi lumen, etc. (Table 5) In particular, PTGS2, NOS3, HMOX1, TNF, ICAM1, and CASP3 are the most significant. (Fig. 6).

**Table 5:** Enriched GO (CC) pathways of Safflower and Salvia compounds in MI and CI samples

Description	***P*** value	p.adjust	qvalue	count
Membrane raft	1.35E-06	3.62E-05	2.56E-05	6
Membrane microdomain	1.38E-06	3.62E-05	2.56E-05	6
Membrane region	1.70E-06	3.62E-05	2.56E-05	6
Caveola	0.000116	0.001863	0.001318	3
Plasma membrane raft	0.000229	0.002933	0.002074	3
Endoplasmic reticulum lumen	0.000435	0.004642	0.003283	4
Platelet alpha granule lumen	0.003048	0.02787	0.019711	2
External side of plasma membrane	0.005049	0.038598	0.027298	3
Platelet alpha granule	0.005428	0.038598	0.027298	2
Golgi lumen	0.007051	0.045123	0.031913	2

To reflect the relationship among those KEGG terms, we reconstructed the GO interaction network using the clusterProfiler package.

### Pathways of Chinese patent medicine

We retrieved Chinese patent medicines including Salvia and Safflower in the Pharmacopoeia of the People’s Republic of China and the National Standard of Chinese Patent Medicines (Table 6). Three kinds of Chinese patent medicines that simultaneously treat MI and CI were identified, including the Xin NaoKang capsule, the Le Mai granule, and the Huoxue Tongmai tablet. The KEGG pathways for those Chinese patent medicines were then found in BATMAN. The enriched KEGG pathway of these four drugs in the treatment of cardiovascular and cerebrovascular diseases was intermingled with the 85 KEGG pathways enriched by Salvia and safflower. It was also determined that the pathways included those in which Salvia and Safflower played a major role in the proprietary Chinese medicines.

**Table 6:** Chinese patent medicines containing Safflower and Salvia

Source	Name	Prescription	Efficacy;
Pharmacopoeia of the People’s Republic of China	Xin Naokang capsule (XNKJN) [20]	Salviae Miltiorrhizae Radix et Rhizoma, Paeoniae Radix Rubra., Polygoni Multiflori Radix, Lycii Fructus, Puerariae Lobatae Radix, Chuanxiong Rhizoma, Carthami Flos, Alismatis Rhizoma, Cyathulae Radix, Pheretima, Curcumae Radix, Polygalae Radix, Anemone altaica Fisch., Ziziphi Spinosae Semen, Deer’s Heart, Glycyrrhizae Radix et Rhizoma	Coronary heart disease angina pectoris, cerebral arteriosclerosis
Pharmacopoeia of the People’s Republic of China	Le Mai Granule (LMKL) [21]	Salviae Miltiorrhizae Radix et Rhizoma, Chuanxiong Rhizoma, Paeoniae Radix Rubra, Carthami Flos, Cyperi Rhizoma, Aucklandiae Radix, Crataegi Fructus	Coronary heart disease, angina pectoris, multiple cerebral infarction
Pharmacopoeia of the People’s Republic of China	Li NaoXin Capsule [22]	Salviae Miltiorrhizae Radix et Rhizoma, Chuanxiong Rhizoma, Puerariae Lobatae Radix, Pheretima, Paeoniae Radix Rubra, Carthami Flos, Curcumae Radix, Polygoni Multiflori Radix, Alismatis Rhizoma, Lycii Fructus, Ziziphi Spinosae Semen, Polygalae Radix, Anemone altaica Fisch., Cyathulae Radix, Glycyrrhizae Radix et Rhizoma	Coronary heart disease, myocardial infarction, cerebral arteriosclerosis, cerebral thrombosis
Pharmacopoeia of the People’s Republic of China	Huoxue Tongmai tablets (HXTMP) [23]	Spatholobi Caulis, Persicae Semen, Salviae Miltiorrhizae Radix et Rhizoma, Paeoniae Radix Rubra, Carthami Flos, Dalbergia odorifera T.Chen, Curcumae Radix, Notoginseng Radix et Rhizoma, Chuanxiong Rhizoma, Citri Reticulatae Pericarpium, Aucklandiae Radix, Acori Tatarinowii Rhizoma, Lycii Fructus, Polygonati Rhizoma, Ginseng Radix et Rhizoma, Ophiopogonis Radix, Borneolum Syntheticum	Coronary heart disease, angina pectoris, cerebral thrombosis, cerebral infarction, sequela of apoplexy, cerebral arteriosclerosis and hyperlipidemia
Standard promulgation of the State Food and Drug Administration	Danhong dripping solution [24]	Salviae Miltiorrhizae Radix et Rhizoma, Carthami Flos	Coronary heart disease, angina pectoris, myocardial infarction, blood stasis type pulmonary heart disease, ischemic encephalopathy, cerebral thrombosis
Standard promulgation of the State Food and Drug Administration	Guan XinJing capsule [25]	Salviae Miltiorrhizae Radix et Rhizoma, Paeoniae Radix Rubra, Chuanxiong Rhizoma, Carthami Flos, Polygonati Odorati Rhizoma, Notoginseng Radix et Rhizoma, Ginseng Radix et Rhizoma, LiquidambarorientalisMill., Borneolum Syntheticum	Chest pain, chest pain, shortness of breath, heart palpitations and coronary heart disease

**Fig. 7:**
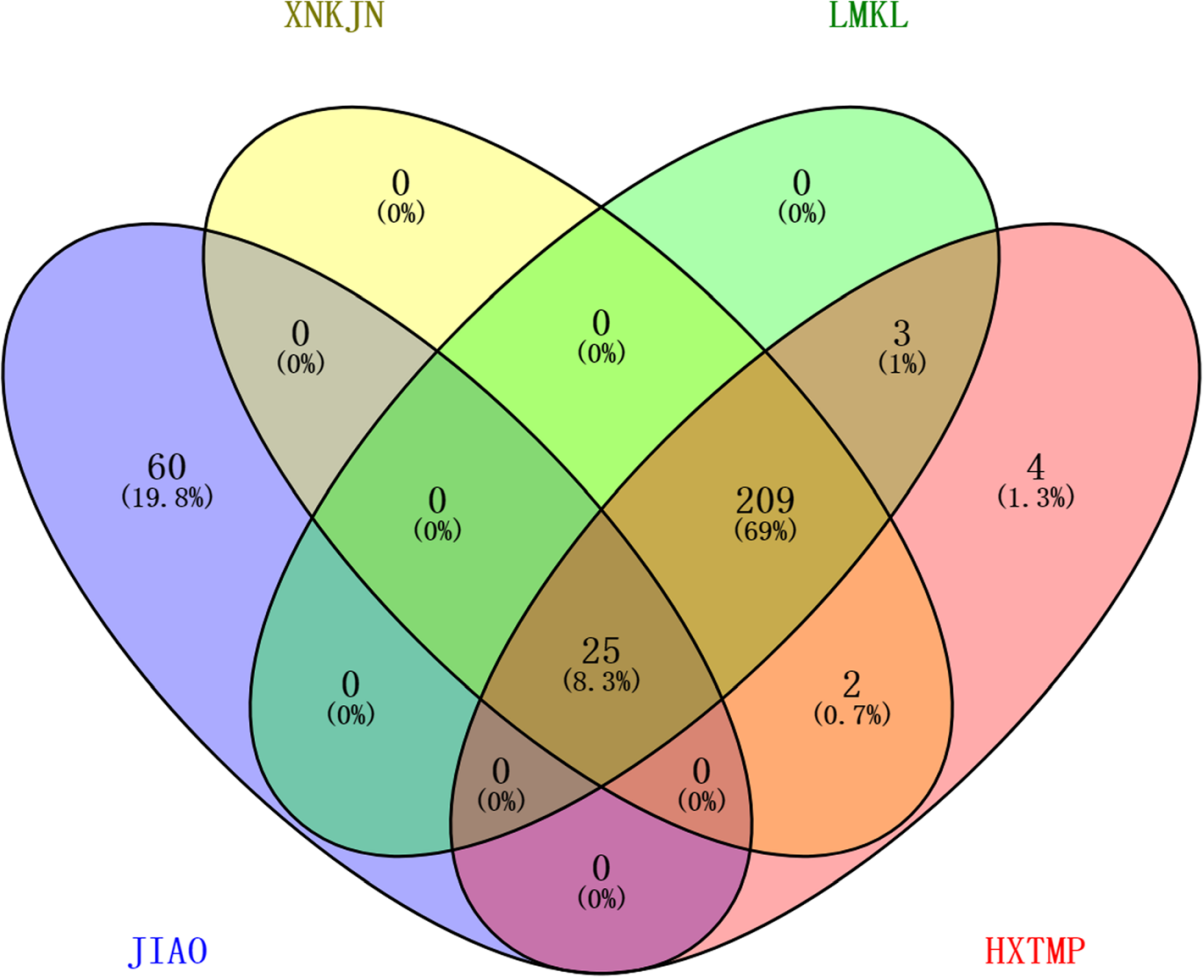
The Venn diagram of common pathways of Chinese patent medicine and Salvia and Safflower. JAIO (represents the common path of Salvia, Safflower, CI, and MI. Eighty-five KEGG pathways were enriched

The results showed that among the four drugs, Salvia and Safflower combined to treat MI and CI in 25 pathways (Fig. 7) (Table 7).The first 10 paths with the most important *P* values are as follows: HIF-1 signaling pathway, TNF signaling pathway, PI3K-AKT signaling pathway, NF-κB signaling pathway, Intestinal immune network for IgA production, Apoptosis, Estrogen signaling pathway, VEGF signaling pathway, P53 signaling pathway, JAK-STAT signaling pathway.

**Table 7:** 25 KEGG pathways for Salvia, Safflower, and Chinese patent medicine in MI and CI samples

No	Description	No	Description
1	HIF-1 signaling pathway	14	Natural killer cell mediated cytotoxicity
2	TNF signaling pathway	15	Ovarian steroidogenesis
3	PI3K-Akt signaling pathway	16	Arginine and proline metabolism
4	NF-kappa B signaling pathway	17	Oxytocin signaling pathway
5	Intestinal immune network for igA production	18	Cytokine-cytokine receptor interaction
6	Apoptosis	19	Steroid hormone biosynthesis
7	Estrogen signaling pathway	20	NOD-like receptor signaling pathway
8	VEGF signaling pathway	21	Retinol metabolism
9	p53 signaling pathway	22	Fc epsilon RI signaling pathway
10	JAK-STAT signaling pathway	23	Focal adhesion
11	MAPK signaling pathway	24	Metabolism of xenobiotics by cytochrome p450
12	Hematopoietic cell lineage	25	Complement and coagulation cascades
13	T cell receptor signaling pathway		

### Pathomorphological observation of brain tissue in rats

**Fig. 8:**
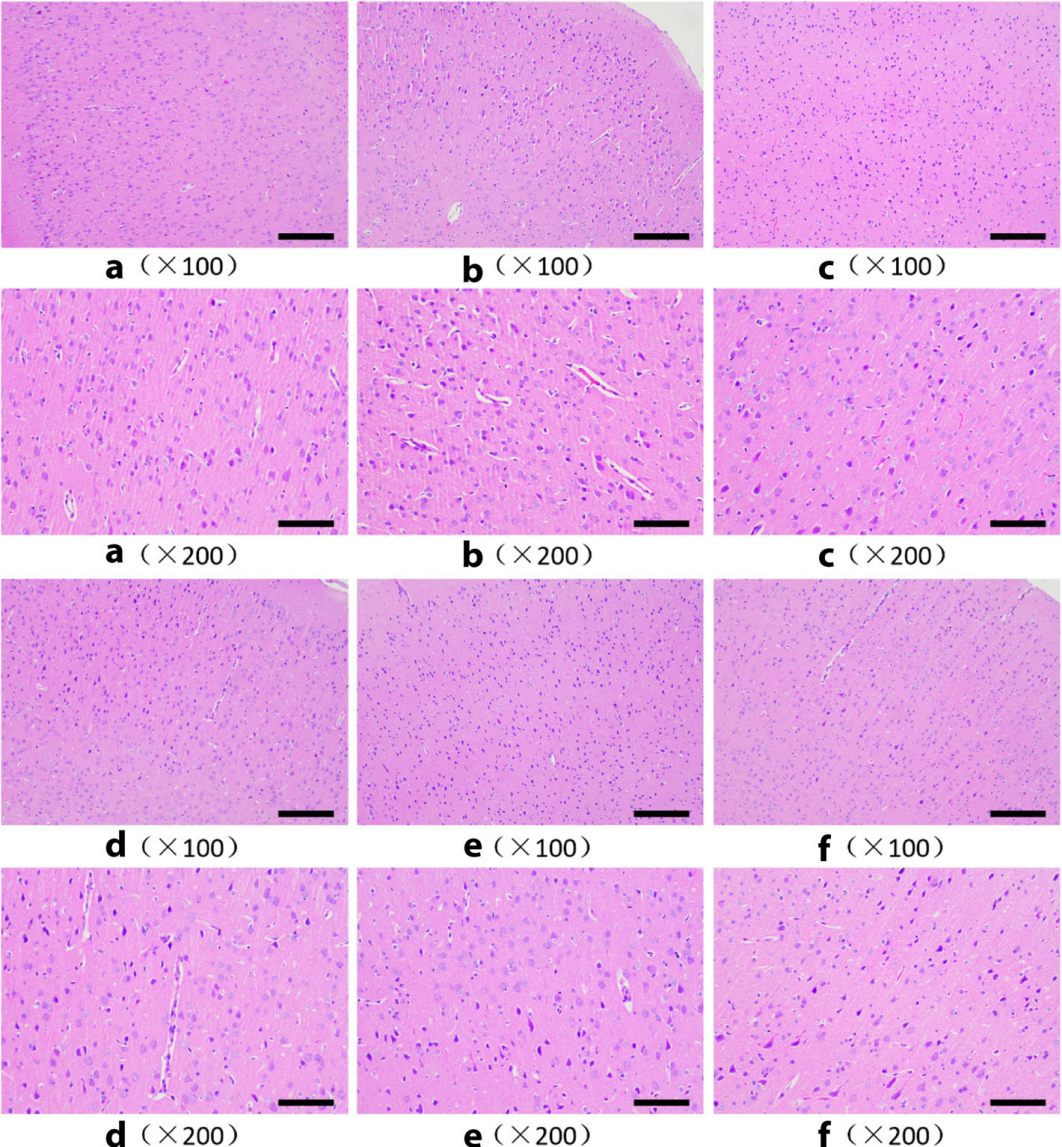
Morphology of nasal mucosa in each group (HE staining, × 100, ×200). **a** Sham operation group. **b** Model group. **c** Nimodipine group. **d** Danhong injection group. (E) Danhong compatibility group. **f** Salvia miltiorrhiza extract group

Figure 8 is a pathological section of brain tissue with focal cerebral ischemic injury in rats. The morphology of the brain tissue cells in the sham-operated group was normal.In the model group, more glial cell proliferation, severe tissue edema, and severe inflammatory cell infiltration could be observed. In the brain tissue cells of the administration group, only a small amount of glial cell proliferation and mild tissue edema was observed.

Observation of the cell morphology of the brain tissue showed that compared with the model group, the four administration groups all had a certain relief effect on the lesions, of which the nimodipine group and the Danhong compatibility group had the best relief inhibition effect.

### Evaluation of neurobehavior and cerebral infarction rate in rats

The results of the T-test showed that compared with the sham operation group, the neurological deficit score (*P < 0.01*) and cerebral infarction rate (*P < 0.05*) of the model group were significantly increased, indicating that the CI model was successful.

**Fig. 9:**
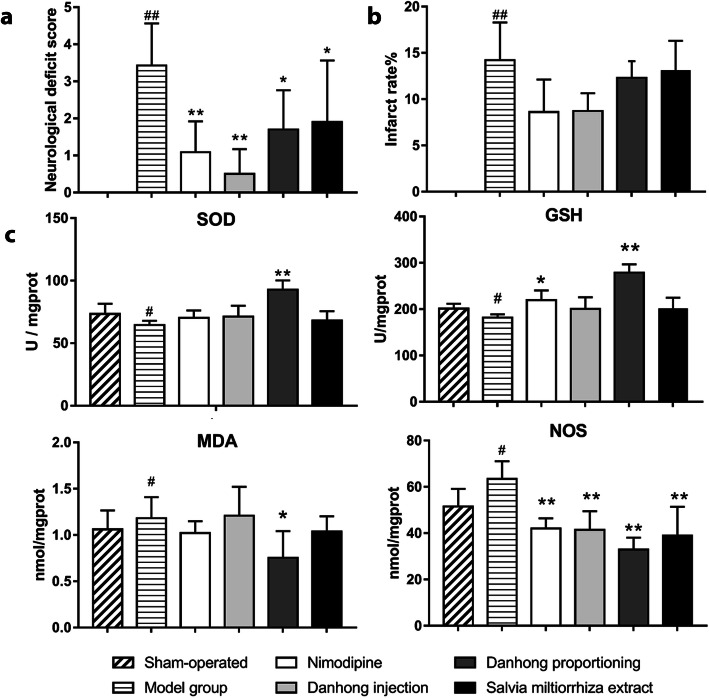
**a** Neurobehavioral Longa scoring system. **b** Determination of cerebral infarction rate. **c** Determination of SOD, GSH-PX, MDA and NOS in serum. (*n* = 6). Compared with the model group, # *P* < 0.05, ## *P* < 0.01 compared with the control group; *P < 0.05, ** *P* < 0.01 compared with the CI group

Compared with the model group, the scores of neurological deficits in the administration group was significantly reduced. The neurological deficit score of Danhong injection group was the lowest (*P < 0.01*), the score of neurological deficit in Nimodipine group (*P < 0.01*) was lower than that in Danhong compatibility group (*P < 0.05*) and Salvia extract group (*P < 0.05*).(Fig. 9a).

The data of the cerebral infarction rate showed that although there was no significant difference in the efficacy of each drug group, the data in the Danhong compatible group was significantly reduced, indicating that the Danhong compatible group was superior to the Danhong injection group and the Danshen extraction group. (Fig. 9b).

### Determination of indicators in rat serum

T-test results show that the levels of serum SOD, MDA, GSH-PX and NOS were measured. As shown in Fig. 9, levels of serum SOD and GSH-PX in the CI model group was significantly decreased compared with that in the sham operation group, while the levels of MDA and NOS were significantly increased.(Table 8).

**Table 8:** Determination of SOD, GSH-PX, MDA and NOS in serum. (*n* = 6) Compared with the model group, # *P* < 0.05, ## *P* < 0.01 compared with the control group; *P < 0.05, ** *P* < 0.01 compared with the CI group

Index	SOD	GSH-PX	MDA	NOS
Sham-operated	73.39 ± 8.17	201.25 ± 10.53	0.94 ± 0.15	51.48 ± 7.66
Model group	64.55 ± 3.41^#^	181.67 ± 7.11^#^	1.18 ± 0.22^#^	63.00 ± 7.65^#^
Nimodipine	70.29 ± 5.85	219.43 ± 20.98*	1.03 ± 0.13	42.02 ± 4.38**
Danhong injection	71.32 ± 8.64	200.43 ± 25.48	1.21 ± 0.31	41.41 ± 8.12**
Danhong proportioning	92.13 ± 7.35**	278.92 ± 17.79**	0.75 ± 0.29*	32.92 ± 5.21**
Salvia miltiorrhiza extract	68.24 ± 7.32	201.20 ± 23.669	1.04 ± 0.16	38.89 ± 12.52**

In indicator GSH-PX, the Danhong compatibility group (*P < 0.01*) and nimodipine group (*P < 0.05*) can significantly increase the content of GSH-PX. In indicators SOD and MDA, only the Danhong compatibility group has significant differences. All administration groups can significantly reduce the content of NOS index (*P < 0.01*). Treatment with Danhong proportioning increased the level of serum SOD and GSH-PX while decreasing the levels of serum MDA and NOS to various exten.The comprehensive effect of the Danhong compatibility group was the best, which was better than that of the Salvia extract group.

According to the comprehensive analysis of the above four groups of evaluation indexes, the treatment group played a role in the treatment of ischemic brain injury by increasing the contents of SOD and GSH and reducing the contents of MDA and NOS. Among them, the Danhong compatibility group had the best therapeutic effect. (Fig. 9c).

## Discussion

It has been reported that many compounds in Safflower and Salvia have therapeutic effects on cardiovascular and cerebrovascular diseases [26]. Safflower yellow has contributed to the decline of blood viscosity and erythrocyte aggregation index, and has anti-coagulation and anti-thrombosis activities in blood stasis rat models [27]. Hydroxysafflor yellow A is known to lengthen coagulation time and relieve cerebral thrombosis induced by cerebral ischemia in mice [28]. Thus, the continual development and application of Safflower are attracting attention for medical purposes [29, 30]. Additionally, extracts of Salvia improved neurological defect scores, increased cerebral blood flow, reduced infarct size, and alleviated brain edema in rats exposed to permanent middle cerebral artery occlusion [31].

Cerebrovascular diseases are characterized by high incidences, disabilities, and mortality. From 1990 to 2000, the annual incidence of stroke in China increased yearly [32]. Approximately 80% of cerebrovascular diseases are acute ischemic stroke caused by acute occlusion of the intracranial artery—also known as CI [33]. MI or acute MI (AMI) occurs when blood flow stops at a part of the heart, causing damage to the heart muscle. The combination of Salvia and Safflower has been reported to be effective in the treatment of MI and CI.

In the present study, 247 genes targeted with 52 compounds of Safflower and 119 genes targeted with 48 compounds of Salvia were identified from the TCMSP database and literature review. Cross-referencing the disease targets with compound targets led to 23 main targets.Those targets were enriched in 85 KEGG pathways. Potential molecular mechanisms of the pathways in which Salvia and Safflower compounds played a major role were identified through the results of the KEGG analyses. Among the four drugs (the Xin NaoKang capsule, the Le Mai granule, and the Huoxue Tongmai tablet), safflower combined with Salvia has 25 KEGG pathways for CI and MI.

The first 10 paths with the most important *P* values are as follows: HIF-1 signaling pathway, TNF signaling pathway, PI3K-AKT signaling pathway, NF-κB signaling pathway, Intestinal immune network for IgA production, Apoptosis, Estrogen signaling pathway, VEGF signaling pathway, P53 signaling pathway, JAK-STAT signaling pathway.

The pathway with the lowest *P*-value was fluid shear stress and atherosclerosis. Atherosclerosis is a complex metabolic disorder that endangers human health. Moreover, atherosclerosis is an important physiological and pathological basis for many ischemic cardiovascular and cerebrovascular diseases [34]. This study partially revealed the effect of Salvia on atherosclerosis. Tanshinone IIA can decrease the level of nitric oxide and increase the activity of superoxide dismutase in human umbilical vein endothelial cells and protect endothelial function and antioxidation [35]. Additionally, tanshinone IIB inhibited the binding of U937 to human aortic endothelial cells mediated by TNF-alpha stimulation [36–38]; Tanshinone IIB enhanced angiogenesis in endothelial cells by up-regulating VEGF and VEGF receptors [39–42]. Studies of the Safflower yellow group also showed that such treatments decrease serum total cholesterol, superoxide dismutase, and propylene glycol. Thus, Safflower yellow can be used to treat atherosclerosis by reducing blood lipid levels and improving antioxidant capacity [43].

Hypoxia-inducible factor-1 (HIF-1) is a transcription factor found widely in mammals and humans under hypoxia. It is induced by changes in molecular oxygen levels in tissues and can activate the expression of many hypoxia-responsive genes. It is a key transcription factor in animals and humans to maintain the stability of the internal environment under hypoxia.

Over the years, the role of HIF-1 alpha in cerebral ischemia and hypoxia has attracted increasing attention. By knocking out the HIF-1a gene, Helton found that HIF-1a reduced the expression of inflammatory mediators such as COX-2, tumor necrosis factor-alpha (TNF-alpha), and IL family proteins following hypoxic-ischemic brain injury [44]. Those studies also indicated that Fufang Danshen tablets had protective effects on brain cells during chronic cerebral ischemia. In addition, Fufang Danshen tablets may initiate the transcription of HIF-1α, which may, in turn, initiate the transcription of EPO, VEGF, and GLUT3. The reduction of brain damage through the anti-hypoxia effect occurred via glycolytic enzymes and their products [45]. Hydroxysafflor yellow A (HSYA) increases the expression of HIF-1α and cell proliferation under hypoxia, which may occur by inhibiting the down-regulation of HIV-1α, VHL, and p53 [46].

Tumor necrosis factor (TNF) is an important inflammatory cytokine in atherosclerosis. Our results show that the TNF-α–308 and TNF-β + 252 loci play important roles in the etiopathogenesis of CI [47].

Tumor necrosis factor-α (TNF-α) antagonism alleviates myocardial ischemia-reperfusion (MI/R) injury [48]. The molecular mechanism of CI injury is complex and involves the inflammatory and apoptotic reactions mediated by interleukin-1 and caspase-3 [49]. Both Danhong injection and HSYA significantly inhibited the over-expression of IL-1beta, TNF-alpha, and caspase-3 genes in brain tissues of CI rats.

The phosphatidylinositol 3′ -kinase(PI3K)-Akt signaling pathway [50] is activated by many types of cellular stimuli or toxic insults and regulates fundamental cellular functions such as transcription, translation, proliferation, growth, and survival. The dysregulation of Akt leads to diseases of major unmet medical needs such as cancer, diabetes, cardiovascular and neurological diseases [51]. The methanol extract of Salvia, are the active compounds as showed by their ability to induce apoptosis through the mitochondrial pathway of apoptosis and PTEN-mediated inhibition of PI3K/Akt pathway [52]. The phosphoinositide 3-kinase (PI3K)/Akt signaling pathway has been reported to be involved in modulating BBB permeability and in isoflurane induced neuroprotection [53].

It is believed that CI can cause the emergency contraction of blood vessels, reduce the blood flow in the cerebral ischemia area, and cause the related symptoms. At this time, the activity of NOS is increased, and the body reacts to produce NO. NO mainly acts on vascular smooth muscle, resulting in vasodilation, decrease of vascular tension and decrease of local vascular tension. The local cerebral blood flow increased, maintained cerebral blood flow and played a certain neuroprotective role [54].

GSH-Px is directly involved in the removal of H_2_O_2_ in the cytoplasm and is vulnerable to the attack of oxygen free radical O^− 2^. The oxygen free radicals produced after ischemia consume a lot of SOD, which makes the content of SOD decrease and also consumes a lot of GSH-Px.

Studies have shown that one of the important mechanisms of ischemic brain damage is due to the accumulation of free radicals and the lipid peroxidation chain reaction mediated by them, resulting in a large number of MDA, resulting in the destruction of membrane structure and function, resulting in neurotoxic damage, resulting in neurological dysfunction [55]. Therefore, the content of MDA in peripheral blood increased, and with the prolongation of ischemia time, the activity of SOD increased gradually, and the content of MDA decreased gradually [56, 57]. Obviously, these targets are considered as potential markers and may play an important role in the treatment of CI.

In this experiment, the rats were given prophylactic administration and the model was made by the MACO method. According to the score of a neurological deficit, the model was made successfully. The cerebral infarction rate, biochemical index, and cytopathological injury were used as evaluation indexes. The therapeutic effects of the Sham operation group, Model group, Nimodipine group, Danhong injection group, Danhong compatibility group, and Salvia extract group were compared. Danhong compatibility group can significantly reduce the cerebral infarction rate, cerebral cell edema, glial cell proliferation, and inflammatory cell infiltration, increase the content of SOD and GSH, and reduce the content of MDA and NOS. The comprehensive effect of the Danhong compatibility group was the best, which was better than that of the Salvia extract group. The results showed that Salvia and safflower extract had a good therapeutic effect on cerebral ischemia through compatibility and interaction.

## Conclusions

In summary, 23 targets of Salvia combined with Safflower in the treatment of CI and MI were identified. This study proposed and applied a network pharmacology-based analysis to suggest that Salvia and Safflower may attenuate CI and MI by regulating targets in the HIF-1 signaling pathway. Four indicators were chosen based on network pharmacology, namely SOD, GSH, MDA and NOS, which have been validated in vivo as potential target of Salvia combined with Safflower for its therapeutic effect on CI. Through the treatment of MCAO model, it provides preliminary evidence for the pharmacological mechanism of Salvia combined with Safflower in the treatment of CI.Danhong compatibility group can significantly reduce the cerebral infarction rate, cerebral cell edema, glial cell proliferation, and inflammatory cell infiltration, increase the content of SOD and GSH, and reduce the content of MDA and NOS.

It is revealed that Salvia miltiorrhiza and Safflower can increase local cerebral blood flow by dilating blood vessels, reduce neurotoxic damage, and protect brain tissue from free radical damage.

## Data Availability

All data are included in this manuscript and in the additional files. Datasets supporting the conclusions of this article are available as a public database from TCMSP, TCM Database@Taiwan, UniPort, Drugbank, TTD, DisGeNET, KEGG, BATMAN-TCM, and ChemMapper.

## Abbreviations

Safflower: *Carthamus tinctorius* L
Salvia: *Salvia miltiorrhiza* Burge
TCMSP: Traditional Chinese Medicine Systems Pharmacology Database and Analysis Platform
MF: Molecular function
BP: Biological processes
PTGS2: Prostaglandin-endoperoxide synthase 2
F2: Coagulation factor II, thrombin
PPARG: Peroxisome proliferator activated receptor gamma
VEGFA: Vascular endothelial growth factor A
IL10: Interleukin 10
IL6: Interleukin 6
TP53: Tumor protein p53
ICAM1: Heme oxygenase 1
HGF: Hepatocyte growth factor
NOS2: Nitric oxide synthase 2
BCL2: BCL2, apoptosis regulator
CYP1A1: Cytochrome P450 family 1 subfamily A member 1
SOD: Superoxide dismutase
GSH-PX: Glutathione peroxidase
CI: Cerebral infarction
TTD: Therapeutic Target Database
GO: Gene Ontology
CC: Cellular components
KEGG: Kyoto Encyclopedia of Genes and Genomes
NR3C2: Nuclear receptor subfamily 3 group C member 2
ESR1: Estrogen receptor 1
PIK3CG: Phosphatidylinositol-4,5-bisphosphate 3-kinase catalytic subunit gamma
MMP9: Matrix metallopeptidase 9
TNF: Tumor necrosis factor
CASP3: Caspase 3
HMOX1: Heme oxygenase 1
SOD1: Superoxide dismutase 1
NOS3: Nitric oxide synthase 3
F7: Coagulation factor VII
CYP3A4: Cytochrome P450 family 3 subfamily A member 4
IL4: Interleukin 4
MDA: Malondialdehyde
NO: Nitric oxide

## References

[1] Meng C, Piwen Z, Yanlin S, Liping S (2012). Advances in pharmacological function of Carthamus tinctorius and its essential constitutes. Glob Tradit Chin Med.

[2] Le-Le Z, Ke T, Zheng-Hai T, Xiao-Jia C, Zhao-Xiang B, Yi-Tao W (2016). Phytochemistry and pharmacology of *Carthamus tinctorius* L. Am J Chin Med.

[3] Shan-yong YI, Li-li GUAN, Jing YANG, Hai-yan LI, Xiao-kun LI, Chao JIANG (2015). HUANG, research advances in pharmacological function and development and application of Carthamus tinctoriu L. Nor Horticul.

[4] Li ZM, Xu SW, Liu PQ (2018). Salvia miltiorrhizaBurge (Danshen): a golden herbal medicine in cardiovascular therapeutics. Acta Pharmacol Sin.

[5] Pang H, Wu L, Tang Y, Zhou G, Qu C, Duan JA (2016). Chemical analysis of the herbal medicine Salviae miltiorrhizae radix et Rhizoma (Danshen). Molecules.

[6] Su CY, Ming QL, Rahman K, Han T, Qin LP (2015). Salvia miltiorrhiza: traditional medicinal uses, chemistry, and pharmacology. Chin J Nat Med.

[7] Wang JF, Wei DQ, Chou KC (2008). Drug candidates from traditional chinese medicines. Curr Top Med Chem.

[8] Cao W, Guo XW, Zheng HZ, Li DP, Jia GB, Wang J (2012). Current progress of research on pharmacologic actions of salvianolic acid B. Chin J Integr Med.

[9] X Z (2005). On simultaneous treatment of heart and brain. Chin Arch Tradit Chin Med.

[10] Pang SC, Zhang JP (2012). Theory and application of simultaneous treatment of heart and brain. J Tradit Chin Med.

[11] Chen D, Tang SH, Lu P, Yang HJ (2015). Mechanism of “treating heart and brain with same methods” based on data science. Zhongguo Zhong Yao Za Zhi.

[12] Hu T, Wei G, Xi M, Yan J, Wu X, Wang Y (2016). Synergistic cardioprotective effects of Danshensu and hydroxysafflor yellow a against myocardial ischemia-reperfusion injury are mediated through the Akt/Nrf2/HO-1 pathway. Int J Mol Med.

[13] Deng JG, Zhang DW, Li J (2011). Experimental study on the effects of water soluble components and compatibility of salvia miltiorrhiza and safflower on myocardial ischemia / reperfusion injury in rats. Chin J Chin Mater Med.

[14] Zhang L, Liu JG, Shi DZ (2009). The protection of active component injection of Slauia miltiorrhiza Bge .And *Carthamus tinctorius* L .On myocardial Ischemical reperfusion injury in rats. Chin J Exp Tradit Med Formulae.

[15] Gao LN, Cui YL, Yan K, Qiu C (2016). Advances in studies on compatibility of Salviae Miltiorrhizae radix et Rhizoma and Carthamii Flos. Chin Tradit Herbal Drugs.

[16] Committee of National Pharmacopoeia (2015). Pharmacopoeia of People’s republic of China.

[17] Yu G, Wang LG, Han Y, He QY (2012). clusterProfiler: an R package for comparing biological themes among gene clusters. OMICS.

[18] Alexa A, Rahnenfuhrer J (2006). topGO: enrichment analysis for gene ontology. R package version.

[19] Ru J, Li P, Wang J, Zhou W, Li B, Huang C (2014). TCMSP: a database of systems pharmacology for drug discovery from herbal medicines. Aust J Chem.

[20] Committee of National Pharmacopoeia. Pharmacopoeia of People’s Republic of China. Beijing: China Medical Science and Technology Press; 2015. Part 1, 727.

[21] Committee of National Pharmacopoeia. Pharmacopoeia of People’s Republic of China. Beijing: China Medical Science and Technology Press; 2015. Part 1, 804.

[22] Committee of National Pharmacopoeia. Pharmacopoeia of People’s Republic of China. Beijing: China Medical Science and Technology Press; 2015. Part 1, 972.

[23] Committee of National Pharmacopoeia. Pharmacopoeia of People’s Republic of China. Beijing: China Medical Science and Technology Press; 2015. Part 1, 1296.

[24] National Medical Prouducts Administration. Standard promulgation of the State Food and Drug Administration. Beijing, 2017; ZGB2017-3.

[25] National Medical Prouducts Administration. Standard promulgation of the State Food and Drug Administration. Beijing, 2012; ZD-0487.

[26] Shi M, Huang F, Deng C, Wang Y, Kai G. Bioactivities, biosynthesis and biotechnological production of phenolic acids in Salvia miltiorrhiza. Crit Rev Food Sci Nutr. 2018:1–12. 10.1080/10408398.2018.1474170 29746788

[27] Li HX, Han SY, Wang XW, Ma X, Zhang K, Wang L, et al. Effect of the carthamins yellow from Carthamus tinctorius L. on hemorheological disorders of blood stasis in rats. Food Chem Toxicol. 2009;47(8):1797–802. 10.1016/j.fct.2009.04.026 19406191

[28] Asgarpanah J, Kazemivash N. Phytochemistry, pharmacology and medicinal properties of Carthamus tinctorius L. Chin J Integr Med. 2013;19(2):153–9. 10.1007/s11655-013-1354-5 23371463

[29] Liu L. Overview of Carthamus tinctorius L. Guangzhou Chemical Industry. 2017;45(12):23–5+68.

[30] Zhang LL, Tian K, Tang ZH, Chen XJ, Bian ZX, Wang YT, et al. Phytochemistry and Pharmacology of Carthamus tinctorius L. Am J Chin Med. 2016;44(2):197–226. 10.1142/S0192415X16500130 27080938

[31] Fei YX, Wang SQ, Yang LJ, Qiu YY, Li YZ, Liu WY, et al. Salvia miltiorrhiza Bunge (Danshen) extract attenuates permanent cerebral ischemia through inhibiting platelet activation in rats. J Ethnopharmacol. 2017;207:57–66. 10.1016/j.jep.2017.06.023 28645780

[32] Yang G, Wang Y, Zeng Y, Gao GF, Liang X, Zhou M, et al. Rapid health transition in China, 1990-2010: findings from the Global Burden of Disease Study 2010. Lancet. 2013;381(9882):1987–2015. 10.1016/S0140-6736(13)61097-1 PMC7159289 23746901

[33] Jauch EC, Saver JL, Adams HP, Bruno A, Connors JJ, Demaerschalk BM, et al. Guidelines for the early management of patients with acute ischemic stroke: a guideline for healthcare professionals from the American Heart Association/American Stroke Association. Stroke. 2013;44(3):870–947. 10.1161/STR.0b013e318284056a 23370205

[34] Moore KJ, Tabas I. Macrophages in the pathogenesis of atherosclerosis. Cell. 2011;145(3):341–55. 10.1016/j.cell.2011.04.005 PMC3111065 21529710

[35] Han JY, Fan JY, Horie Y, Miura S, Cui DH, Ishii H, et al. Ameliorating effects of compounds derived from Salvia miltiorrhiza root extract on microcirculatory disturbance and target organ injury by ischemia and reperfusion. Pharmacol Ther. 2008;117(2):280–95. 10.1016/j.pharmthera.2007.09.008 18048101

[36] Che Y, Ruan Y, Li L, Chu Y, Xu X, Wang Q, et al. Effects of Salvia miltiorrhiza extracts on rat hypoxic pulmonary hypertension, heme oxygenase-1 and nitric oxide synthase. Chin Med J. 2003;116(5):757–60. 12875696

[37] Wang J, Xiong X, Feng B. Cardiovascular effects of salvianolic Acid B. Evid Based Complement Alternat Med. 2013;2013:247948. 10.1155/2013/247948 PMC3691933 23840250

[38] He X, Shen Q. Salvianolic acid B promotes bone formation by increasing activity of alkaline phosphatase in a rat tibia fracture model: a pilot study. BMC Complement Altern Med. 2014;14:493. 10.1186/1472-6882-14-493 PMC4301899 25510675

[39] Lay IS, Chiu JH, Shiao MS, Lui WY, Wu CW. Crude extract of Salvia miltiorrhiza and salvianolic acid B enhance in vitro angiogenesis in murine SVR endothelial cell line. Planta Med. 2003;69(1):26–32. 10.1055/s-2003-37034 12567275

[40] Duval F, Moreno-Cuevas JE, González-Garza MT, Rodríguez-Montalvo C, Cruz-Vega DE. Protective mechanisms of medicinal plants targeting hepatic stellate cell activation and extracellular matrix deposition in liver fibrosis. Chin Med. 2014;9(1):27. 10.1186/s13020-014-0027-4 PMC4299307 25606051

[41] Yang Y, Ge PJ, Jiang L, Li FL, Zhu QY. Modulation of growth and angiogenic potential of oral squamous carcinoma cells in vitro using salvianolic acid B. BMC Complement Altern Med. 2011;11:54. 10.1186/1472-6882-11-54 PMC3158556 21726465

[42] Chang TM, Shi GY, Wu HL, Wu CH, Su YD, Wang HL, et al. Effects of salvianolic Acid B on protein expression in human umbilical vein endothelial cells. Evid Based Complement Alternat Med. 2011;2011:213050. 10.1155/2011/213050 PMC3057447 21423689

[43] Wei ZY, Xu WJ, Dong JJ. Studies on hydroxysafflower yellow A repairing the metabolic disturbances of early atherosclerosis based on fatty acid profiling. Acta Pharmaceutica Sinica. 2018:1–9.

[44] Webster KA, Graham RM, Thompson JW, Spiga MG, Frazier DP, Wilson A, et al. Redox stress and the contributions of BH3-only proteins to infarction. Antioxid Redox Signal. 2006;8(9-10):1667–76. 10.1089/ars.2006.8.1667 16987020

[45] Zhu YL, Liu QQ, Yang WQ (2005). Effects of compound tablet of red sage root on the expression of HIF -1αin brain of rat during chronic cerebral ischemia. J Practical Nerv Dis..

[46] Lian Q, Zhao DL, Zhu HB (2008). Hydroxysafflor yellow A up-regulates HIF-1α viainhibition of VHL and p53 in Eahy926 cell line exposed to hypoxi. Acta Pharmaceutica Sinica..

[47] Um JY, An NH, Kim HM (2003). TNF-alpha and TNF-beta gene polymorphisms in cerebral infarction. J Mol Neurosci..

[48] Gao C, Liu Y, Yu Q, Yang Q, Li B, Sun L (2015). TNF-α antagonism ameliorates myocardial ischemia-reperfusion injury in mice by upregulating adiponectin. Am J Physiol Heart Circ Physiol..

[49] Li HH, Lou SJ (2013). Effects of hypoxia on the expression of IL-1β、IL-6 and TNF-α mRNA in cerebral cortex cells of rats. Chin J Neuroanat..

[50] Engelman JA, Luo J, Cantley LC (2006). The evolution of phosphatidylinositol 3-kinases as regulators of growth and metabolism. Nat Rev Genet..

[51] Hers I, Vincent EE, Tavaré JM (2011). Akt signalling in health and disease. Cell Signal..

[52] Ye YT, Zhong W, Sun P, Wang D, Wang C, Hu LM, et al. Apoptosis induced by the methanol extract of Salvia miltiorrhiza Bunge in non-small cell lung cancer through PTEN-mediated inhibition of PI3K/Akt pathway. J Ethnopharmacol. 2017;200(undefined):107–16. 10.1016/j.jep.2016.12.051 28088493

[53] Chi QZ, Mellender SJ, Kiss GK, Liu X, Weiss HR (2017). Blood -brain barrier disruption was less under isoflurane than pentobarbital anesthesia via a PI3K/Akt pathway in early cerebral ischemia. Brain Res Bull..

[54] Lin F, Xiaorong C, Jie Y, Jun Z, Chao W (2008). Effect of electroacupuncture on NO, NOS and ET-1 in serum of rats with acute cerebral ischemia. Journal of Traditional Chinese Medicine University of Hunan..

[55] Jie S, Mingru Y, Yu Z, Xiaoxuan R, Yin L, Dengfang Z, Lufeng Z (2012). Effect of early intervention of Xingnao Kaiqiao acupuncture therapy on SOD, MDA in rats with cerebral ischemia and reperfusion. China Journal of Traditional Chinese Medicine and Pharmacy.

[56] Y I, DM L. The molecular basis of brain injury and brain edema: the role of oxygen free radicals. Neurosurgery. 1990;27(1):1–11. 10.1097/00006123-199007000-00001. . 10.1097/00006123-199007000-00001 2198480

[57] S C, DP R, AP C, D S. Antioxidant therapy: a new pharmacological approach in shock, inflammation, and ischemia/reperfusion injury. Pharmacol Rev. 2001;53(1):135–59. 11171943

